# Supplementary material for: Network Pharmacological Analysis and Experimental Validation of the Effect of Smilacis Glabrae Rhixoma on Gastrointestinal Motility Disorder
Source: Plants (Basel). 2023 Mar 30;12(7):1509. doi: 10.3390/plants12071509 (PMC10096900; doi:10.3390/plants12071509)
Supplement: Supplementary file 1 [file plants-12-01509-s001.zip › plants-2289797-supplementary.pdf]

Table S1 Potential active compounds of *Smilacis Glabrae Rhixoma*

Molecule Name	Structure	MW	OB (%)	Caco-2	DL
(-)-epicatechin	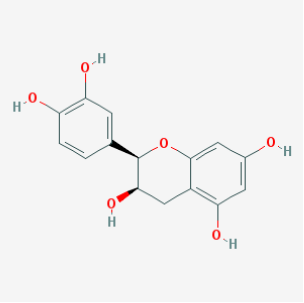	290.29	28.93	-0.03	0.24
(-)-taxifolin	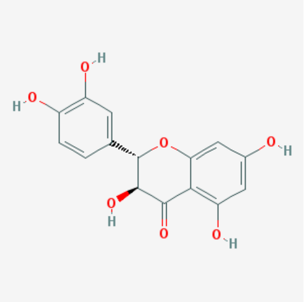	304.27	60.51	-0.24	0.27
()-Terpinen-4-ol	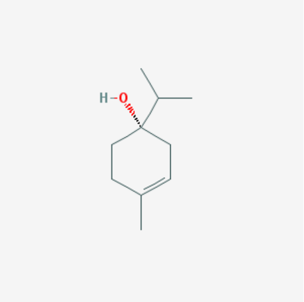	154.28	81.41	1.36	0.03
(2R,3R)-2-(3,5-dihydroxyphenyl)-3,5,7-trihydroxychroman-4-one	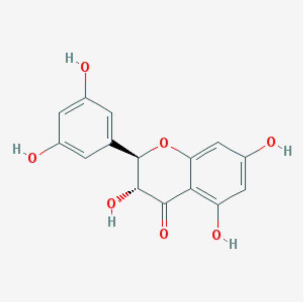	304.27	63.17	-0.34	0.27
(2S,3R)-3,5,7-trihydroxy-2-(4-hydroxyphenyl)chroman-4-one	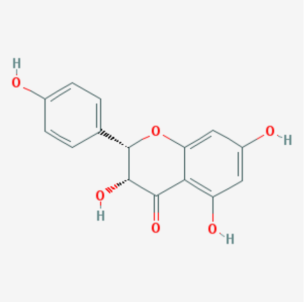	288.27	22.73	-0.1	0.24

Molecule Name	Structure	MW	OB (%)	Caco-2	DL
(L)-alpha-Terpineol	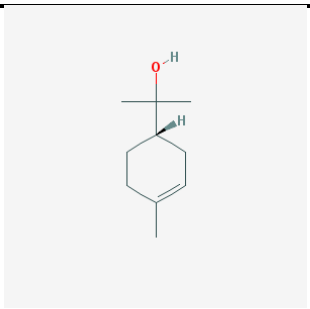	154.28	48.8	1.39	0.03
(R)-linalool	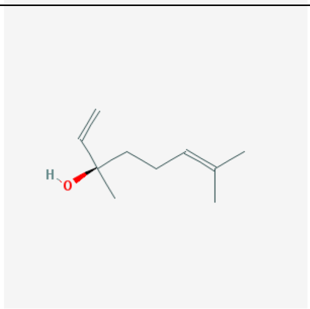	154.28	39.8	1.33	0.02
[(2S,3S,4R,5R)-2-[(2R,3R,4S,5S,6R)-3-acetyloxy-6-(acetyloxymethyl)-4,5-dihydroxyoxan-2-yl]oxy-4-hydroxy-2,5-bis[[(E)-3-(4-hydroxy-3-methoxyphenyl)prop-2-enoyl]oxymethyl]oxolan-3-yl] (E)-3-(4-hydroxy-3-methoxyphenyl)prop-2-enoate	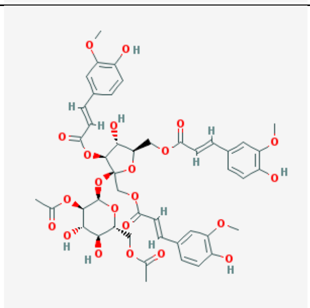	954.96	3.04	-2.17	0.2
2H-3,9a-Methano-1-benzoxepin-9-methanol, octahydro-2,2,5a-trimethyl-, (3R-(3alpha,5aalpha,9alpha,9aalpha))-	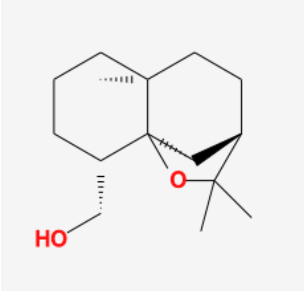	238.41	98.38	1.01	0.14
3-O-caffeoylshikimic acid	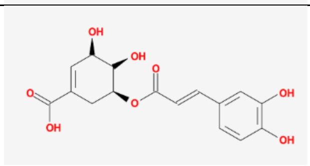	336.32	14.48	-0.81	0.3
4,7-Dihydroxy-5-methoxyl-6-methyl-8-formyl-flavan	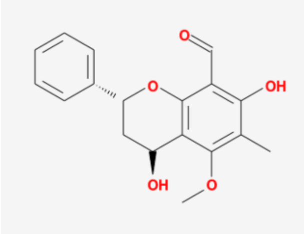	314.36	37.03	0.48	0.28

Molecule Name	Structure	MW	OB (%)	Caco-2	DL
alpha-Eudesmol	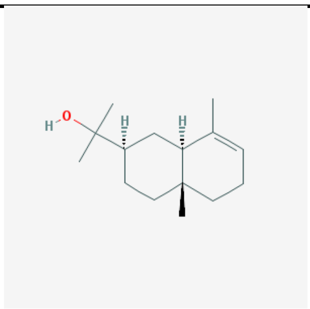	222.41	25.02	1.31	0.1
Aromadedrin	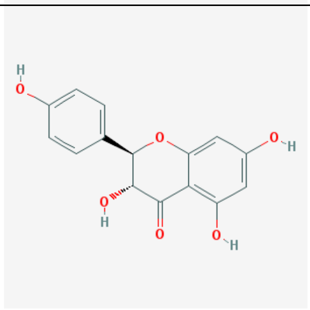	288.27	23.04	-0.08	0.24
astilbin	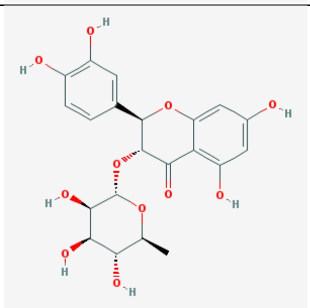	450.43	36.46	-1.29	0.74
beta-Eudesmol	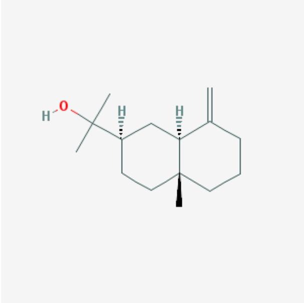	222.41	26.09	1.32	0.1
beta-sitosterol	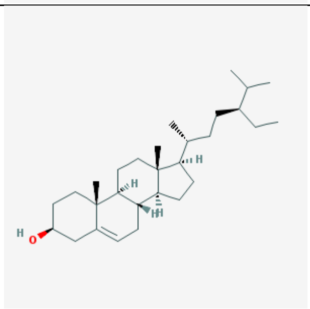	414.79	36.91	1.32	0.75

Molecule Name	Structure	MW	OB (%)	Caco-2	DL
Cedar acid	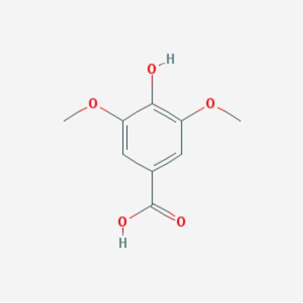	198.19	47.78	0.5	0.06
Cedrol	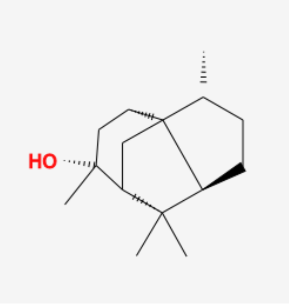	222.41	16.23	1.35	0.12
cis-Dihydroquercetin	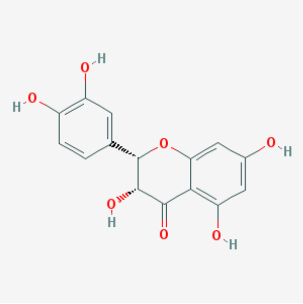	304.27	66.44	-0.34	0.27
delta-amorphene	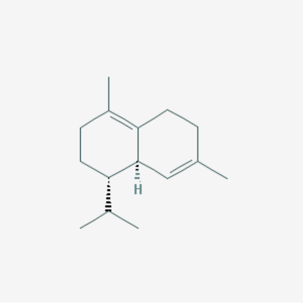	204.39	17.95	1.85	0.08
Dihydro-beta-ionone	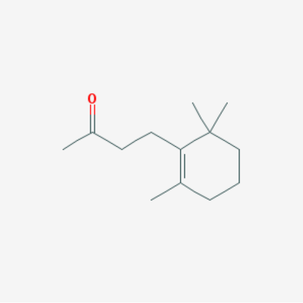	194.35	26.25	1.35	0.05

Molecule Name	Structure	MW	OB (%)	Caco-2	DL
Dihydroresveratrol	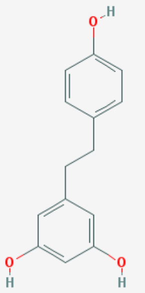	230.28	87.27	0.81	0.11
Dioscin	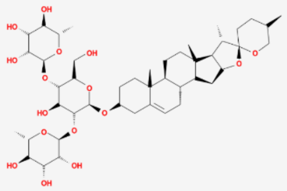	869.17	17.75	-2.19	0.06
diosgenin	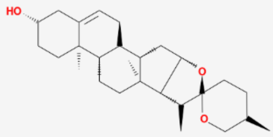	414.69	80.88	0.82	0.81
Dodekan	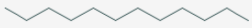	170.38	17.74	1.79	0.02
EIC	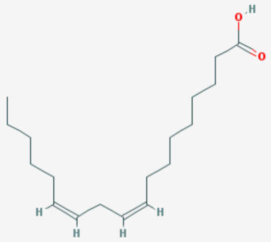	280.5	41.9	1.16	0.14
engeletin	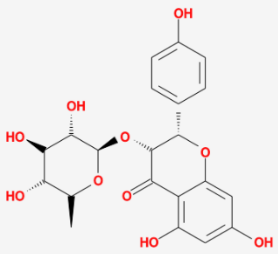	434.43	2.65	-1.01	0.7

Molecule Name	Structure	MW	OB (%)	Caco-2	DL
Enhydrin	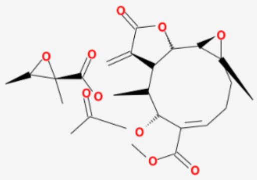	464.51	40.56	-0.36	0.74
FER	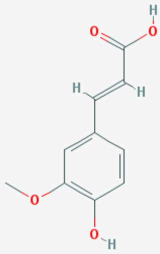	194.2	39.56	0.47	0.06
hexadecane		226.5	12.32	1.81	0.06
HMF	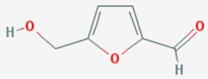	126.12	45.07	0.05	0.02
isoastilbin	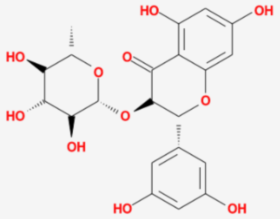	450.43	27.05	-1.19	0.74
isoengelitin	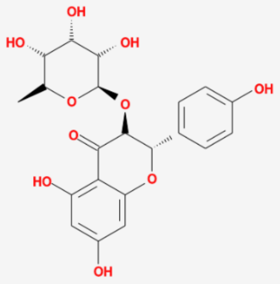	434.43	34.65	-0.91	0.7

Molecule Name	Structure	MW	OB (%)	Caco-2	DL
Isoeruboside_B	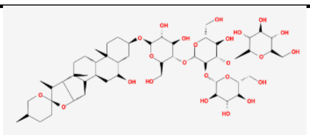	1,081.35	8.88	-3.93	0.02
Isoeruboside_B_qt	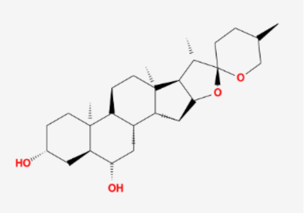	432.71	13.34	0.12	0.79
Istidina	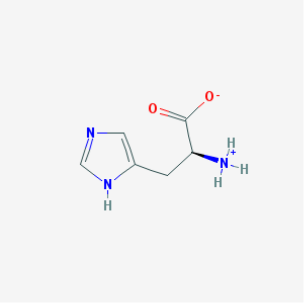	155.18	53.18	-0.25	0.03
L-Bornyl acetate	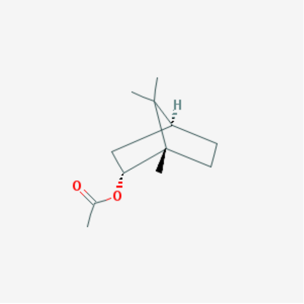	196.32	65.52	1.29	0.08
Methyllinolenate	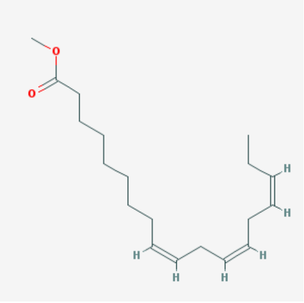	292.51	46.15	1.48	0.17
myristic acid	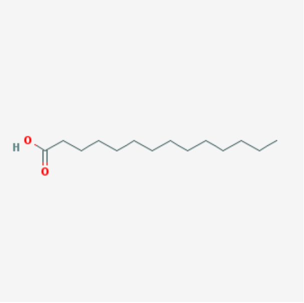	228.42	21.18	1.07	0.07

Molecule Name	Structure	MW	OB (%)	Caco-2	DL
MYS		212.47	13.98	1.81	0.05
naringenin	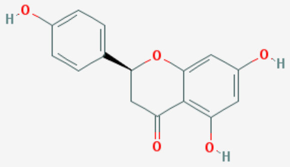	272.27	59.29	0.28	0.21
n-butyl-α-D-fructoufranoside	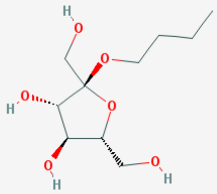	236.3	8.53	-0.85	0.08
n-butyl-β-D-fructopyronoside	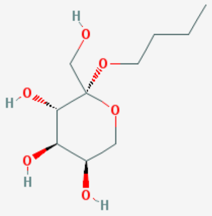	236.3	18.17	-0.56	0.08
n-butyl-β-D-fructoufranoside	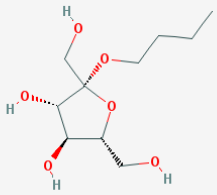	236.3	9.94	-0.78	0.08

Molecule Name	Structure	MW	OB (%)	Caco-2	DL
NCA	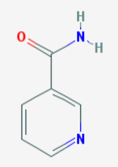	122.14	71.13	0.44	0.02
Neoastilbin	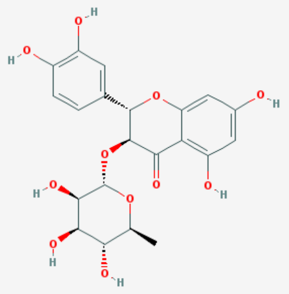	450.43	40.54	-1.07	0.74
nonane	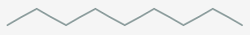	128.29	29.23	1.75	0.01
octacosanal	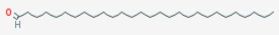	408.84	12.19	1.56	0.42
oleic acid	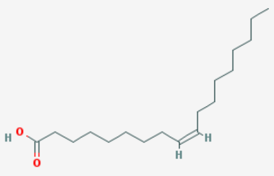	282.52	33.13	1.17	0.14

Molecule Name	Structure	MW	OB (%)	Caco-2	DL
palmitic acid	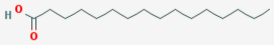	256.48	19.3	1.09	0.1
Palmitone	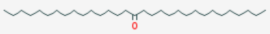	450.93	11.85	1.57	0.48
quercetin	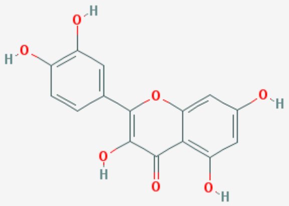	302.25	46.43	0.05	0.28
resveratrol	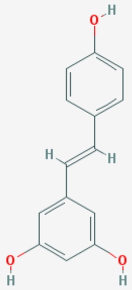	228.26	19.07	0.8	0.11
Sitogluside	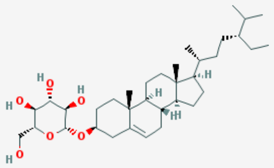	576.95	20.63	-0.14	0.62

Molecule Name	Structure	MW	OB (%)	Caco-2	DL
sitosterol	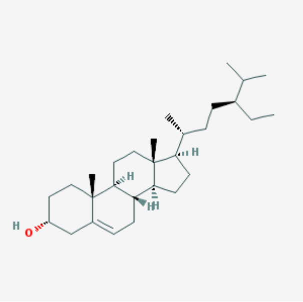	414.79	36.91	1.32	0.75
SKM	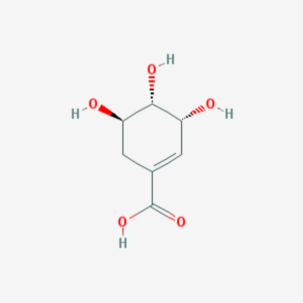	174.17	46.24	-1.16	0.04
Smiglaside C	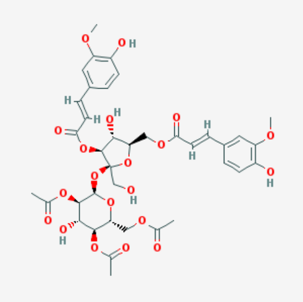	820.82	4.21	-1.86	0.31
Smiglaside D	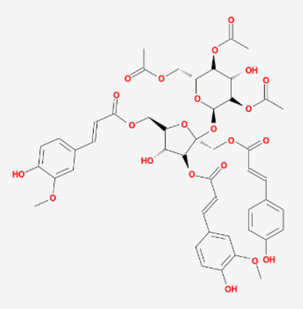	966.97	3.05	-1.93	0.19
Smiglaside E	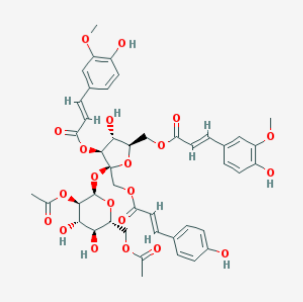	924.93	3.17	-2.06	0.22
Smilagenin	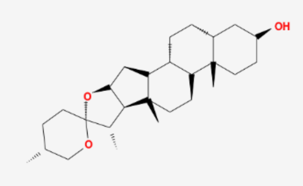	416.71	14.15	0.73	0.81

Molecule Name	Structure	MW	OB (%)	Caco-2	DL
Sodium tauropythocholate	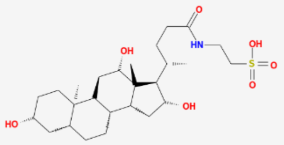	514.78	15.2	-0.68	0.86
stearic acid	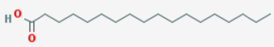	284.54	17.83	1.15	0.14
Stigmasterol	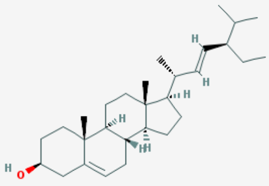	412.77	43.83	1.44	0.76
succinic acid	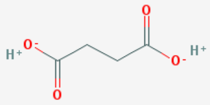	118.1	29.62	-0.44	0.01
Syrionylglycerol-beta-syringaresinol	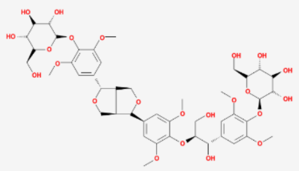	969.05	3.01	-2.86	0.09
Syrionylglycerol-beta-syringaresinol_qt	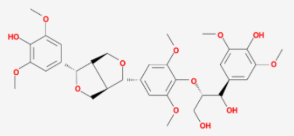	644.73	8.4	-0.56	0.6
Tannin	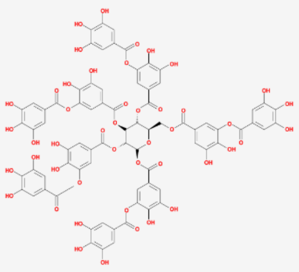	1,701.27	7.89	-6.1	0.03

Molecule Name	Structure	MW	OB (%)	Caco-2	DL
taxifolin	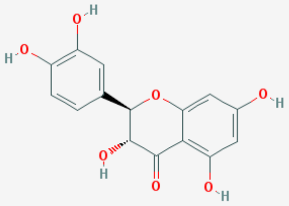	304.27	57.84	-0.23	0.27
trans-2,4-decadienal	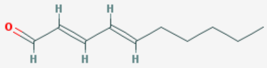	152.26	51.03	1.4	0.02
Tricosane		324.71	8.33	1.85	0.21
Tulipane	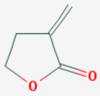	98.11	75.16	1.1	0.01
TWT		310.68	8.37	1.85	0.18

Molecule Name	Structure	MW	OB (%)	Caco-2	DL
ZINC00968101	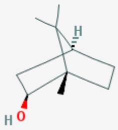	154.28	88.03	1.27	0.05
β-terpineol	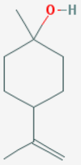	154.28	47.97	1.19	0.03

Table S2 Target genes of *Smilacis Glabrae Rhixoma*

Target name	Gene Name	UniProt ID
1-aminocyclopropane-1-carboxylate deaminase	acdS	P30297
26S proteasome non-ATPase regulatory subunit 3	PSMD3	O43242
2-isopropylmalate synthase	LEU4	P06208
3-hydroxy-3-methylglutaryl-coenzyme A reductase	HMGCR	P04035
3-oxoacyl-[acyl-carrier-protein] synthase 1	OXSM	Q9NWU1
40S ribosomal protein S6	RPS6	P62753
4-aminobutyrate aminotransferase, mitochondrial	ABAT	P80404
5'-AMP-activated protein kinase subunit gamma-2	PRKAG2	Q9UGJ0
5-hydroxytryptamine 2A receptor	HTR2A	P28223
5-hydroxytryptamine receptor 3A	HTR3A	P46098
72 kDa type IV collagenase	MMP2	P08253
78 kDa glucose-regulated protein	HEL-S-89n	V9HWB4
Acetylcholinesterase	ACHE	P22303
Acetyl-CoA acetyltransferase	ACAT1	P24752
Acetyl-CoA carboxylase 1	ACACA	Q13085
Activator of 90 kDa heat shock protein ATPase homolog 1	AHSA1	O95433
Acyl-CoA desaturase	FADS2	O95864
Adenylosuccinate synthetase	ADSS1	Q8N142
Adiponectin	ADIPOQ	Q15848
Adiponectin receptor protein 1	ADIPOR1	Q96A54
Adiponectin receptor protein 2	ADIPOR2	Q86V24
Alcohol dehydrogenase 1A	ADH1A	P07327
Alcohol dehydrogenase 1B	ADH1	P00330
Alcohol dehydrogenase 1C	ADH1C	A0A087WU<br>C4
Aldo-keto reductase family 1 member C1	AKR1C1	Q04828
Aldose reductase	AKR1B1	P15121
Alpha- and gamma-adaptin-binding protein p34	AAGAB	Q6PD74
Alpha-1A adrenergic receptor	ADRA1A	P35348
Alpha-1B adrenergic receptor	ADRA1B	P11615
Alpha-1D adrenergic receptor	ADRA1D	P25100
Alpha-2A adrenergic receptor	ADRA2A	P08913
Alpha-2B adrenergic receptor	ADRA2B	P18089
Amine oxidase [flavin-containing] A	MAOA	P21397
Amine oxidase [flavin-containing] B	MAOB	P27338
Androgen receptor	AR	P10275

Target name	Gene Name	UniProt ID
Angiotensin-converting enzyme	ACE	P12821
Apolipoprotein B-100	APOB	P04114
Apoptosis regulator BAX	BAX	Q07812
Apoptosis regulator Bcl-2	BCL2	P10415
Apoptotic protease-activating factor 1	APAF1	A7E2A2
Arachidonate 5-lipoxygenase	ALOX5	P09917
Aromatic-amino-acid aminotransferase	tyrB	P04693
Aryl hydrocarbon receptor	AHR	P35869
Aspartate aminotransferase	GOT1	P17174
Aspartate aminotransferase, cytoplasmic	GOT1	P17174
Aspartate aminotransferase, mitochondrial	GOT2	P00505
Aspartyl aminopeptidase	DNPEP	Q9ULA0
ATP-binding cassette sub-family G member 2	ABCG2	F6Q6E8
Bacillolysin	npr	P05806
Baculoviral IAP repeat-containing protein 3	BIRC3	Q13489
Baculoviral IAP repeat-containing protein 4	XIAP	H9FTU3
Baculoviral IAP repeat-containing protein 5	BIRC5	O15392
Basal cell adhesion molecule	BCAM	P50895
Basigin	BSG	P35613
Bcl2 antagonist of cell death	BAD	Q92934
Bcl-2 homologous antagonist/killer	BAK1	Q16611
Bcl-2-binding component 3	BBC3	Q9BXH1
Bcl-2-like protein 1	BCL2L1	Q07817
Bcl-2-like protein 11	BCL2L11	O43521
Bcl-2-related protein A1	BCL2A1	Q16548
BDNF/NT-3 growth factors receptor	NTRK2	Q16620
Beta-1 adrenergic receptor	ADRB1	P07700
Beta-2 adrenergic receptor	ADRB2	P07550
Beta-lactamase	ampC	P00811
Brain-derived neurotrophic factor	BDNF	P23560
Branched chain amino acid-dependent aminotransferase	BCAT1	P54687
Breast cancer type 1 susceptibility protein	BRCA1	P38398
Breast cancer type 2 susceptibility protein	BRCA2	P51587
C5a anaphylatoxin chemotactic receptor	C5AR1	P21730
Calcium-activated potassium channel subunit alpha 1	KCNMA1	Q12791
Calmodulin	CALM	P62157
cAMP-dependent protein kinase inhibitor alpha	PKIA	P61925

Target name	Gene Name	UniProt ID
Canalicular multispecific organic anion transporter 1	ABCC2	Q92887
Canalicular multispecific organic anion transporter 2	ABCC3	O15438
Carbonic anhydrase II	carbonic anhydrase II/ CA II	Q6LD99
CASP8 and FADD-like apoptosis regulator	CFLAR	O15519
Caspase-3	CASP3	P42574
Caspase-8	CASP8	Q14790
Caspase-9	CASP9	P55211
Catalase	CAT	P04040
Catechol O-methyltransferase	COMT	P21964
Catenin beta-1	CTNNB1	P35222
Cathepsin D	CTSD	P07339
Caveolin-1	CAV1	Q03135
Cbp/p300-interacting transactivator 1	CITED1	Q99966
C-C chemokine receptor type 2	CCR2	P41597
C-C motif chemokine 2	CCL2	P13500
CD320 antigen	CD320	Q9NPF0
CD40 ligand	CD40LG	P29965
Cell division control protein 2 homolog	CDC2	P43290
Cell division control protein 42 homolog	CDC42	P60953
Cell division protein kinase 4	CDK4	P11802
Cell division protein kinase 6	CDK6	Q00534
Cell division protein kinase 7	CDC7	O00311
Cell-death-related nuclease 7	crn-7	P34387
Cellular tumor antigen p53	TP53	P04637
CGMP-inhibited 3',5'-cyclic phosphodiesterase A	PDE3A	Q14432
Cholecystokinin	CCK	P06307
Cholesteryl ester transfer protein	CETP	P11597
Choline-phosphate cytidylyltransferase A	PCYT1A	P49585
Cholinesterase	BCHE	P06276
Chymotrypsinogen B	CTRB1	P17538
Claudin-4	CLDN4	O14493
Coagulation factor VII	F7	P08709
Coagulation factor Xa	F10	P00742
Collagen alpha-1(I) chain	COL1A1	P02452
Collagen alpha-1(II) chain	COL2A1	P02458
Collagen alpha-1(III) chain	COL3A1	P02461

Target name	Gene Name	UniProt ID
C-reactive protein	CRP	P02741
CREB/ATF bZIP transcription factor	CREBZF	Q9NS37
CREB-regulated transcription coactivator 2	CRTC2	Q53ET0
C-X-C motif chemokine 10	CXCL10	P02778
C-X-C motif chemokine 11	CXCL11	O14625
C-X-C motif chemokine 2	CXCL2	P19875
Cyclic AMP-responsive element-binding protein 1	CREB1	P16220
Cyclin-dependent kinase inhibitor 1	CDKN1A	P38936
Cyclin-dependent kinase inhibitor 2A, isoforms 1/2/3	CDKN2A	A0A2K5KNH1
Cytochrome P450 19A1	CYP19A1	P11511
Cytochrome P450 1A1	CYP1A1	P04798
Cytochrome P450 1A2	CYP1A2	P05177
Cytochrome P450 1B1	CYP1B1	Q16678
Cytochrome P450 3A4	CYP3A4	P08684
Cytochrome P450-cam	camC	P00183
Cytosolic phospholipase A2	PLA2G4A	P47712
D-alanyl-D-alanine carboxypeptidase	vanYB	Q47746
DDB1- and CUL4-associated factor 5	DCAF5	Q96JK2
Deacetoxycephalosporin C synthetase	cefE	B5GLB0
Diacylglycerol O-acyltransferase 2	DGAT2	Q96PD7
Dipeptidyl peptidase IV	DPP4	P27487
DNA damage-inducible transcript 3 protein	DDIT3	P35638
DNA gyrase subunit B	gyrB	P0AES6
DNA topoisomerase 1	TOP1	P11387
DNA topoisomerase 2-alpha	TOP2A	P11388
DNA topoisomerase II	TOP2A	P11388
Dopamine D1 receptor	DRD1	P21728
Dual oxidase 2	DUOX2	Q9NRD8
Ectonucleotide pyrophosphatase/phosphodiesterase family member 7	ENPP7	Q6UWV6
Endothelin-1	EDN1	P05305
Epidermal growth factor receptor	EGFR	P00533
E-selectin	SELE	P16581
Estrogen receptor	ESR1	P03372
Estrogen sulfotransferase	SULT1E1	P49888
ETS domain-containing protein Elk-1	ELK1	P19419
Eukaryotic translation initiation factor 2 subunit 1	EIF2S1	P05198

Target name	Gene Name	UniProt ID
Eukaryotic translation initiation factor 6	EIF6	P56537
Fatty acid synthase	FASN	P49327
Fatty acid-binding protein, liver	FABP1	P07148
Ferrichrome-iron receptor	fhuA	P06971
Forkhead box protein O1	FOXO1	Q12778
Formate acetyltransferase 1	pflB	P09373
Fumarate reductase flavoprotein subunit	frdA	P00363
G1/S-specific cyclin-D1	CCND1	P24385
G1/S-specific cyclin-D2	CCND2	P30279
G1/S-specific cyclin-E1	CCNE1	P24864
G1/S-specific cyclin-E2	CCNE2	O96020
G2/mitotic-specific cyclin-B1	CCNB1	P14635
Gamma-aminobutyric acid receptor subunit alpha-1	GABRA1	P14867
Gamma-aminobutyric-acid receptor alpha-2 subunit	GABRA2	P47869
Gamma-aminobutyric-acid receptor alpha-3 subunit	GABRA3	P34903
Gamma-aminobutyric-acid receptor alpha-5 subunit	GABRA5	P31644
Gamma-aminobutyric-acid receptor subunit alpha-4	GABRA4	D6RB66
Gamma-aminobutyric-acid receptor subunit alpha-6	GABRA6	Q16445
Gap junction alpha-1 protein	GJA1	P17302
Glucagon	GCG	P01275
Glucarate dehydratase	gudD	P0AES2
Glucocorticoid receptor	NR3C1	P04150
Glucose--fructose oxidoreductase	gfo	Q07982
Glutamate [NMDA] receptor subunit zeta-1	GRIN1	Q05586
Glutamate dehydrogenase 1, mitochondrial	GLUD1	P00367
Glutamate receptor 2	GRIA2	P42262
Glutamate--cysteine ligase catalytic subunit	GCLC	P48506
Glutamyl aminopeptidase	ENPEP	Q07075
Glutathione reductase, mitochondrial	GSR	P00390
Glutathione S-transferase Mu 1	GSTM1	P09488
Glutathione S-transferase Mu 2	GSTM2	P28161
Glutathione S-transferase P	GSTP1	P09211
Glutathione synthetase	GSS	P48637
Glycine receptor alpha-1 chain	GLRA1	P23415
Growth-inhibiting protein 18	GIG18	Q2TU84
GTP cyclohydrolase 1	GCH1	P30793
Haloalkane dehalogenase	linB	D4Z2G1

Target name	Gene Name	UniProt ID
Heat shock factor protein 1	HSF1	Q00613
Heat shock protein beta-1	HSPB1	P04792
Heat shock protein HSP 90	HSP90AA1	P07900
Heme oxygenase 1	HMOX1	P09601
Heparin-binding growth factor 2	FGF2	P09038
Hepatocyte growth factor	HGF	P14210
Hexokinase-2	HK2	P52789
High affinity nerve growth factor receptor	NTRK1	P04629
Homeobox protein Nkx-3.1	NKX3-1	Q99801
Hyaluronan synthase 2	HAS2	Q92819
Hypoxia-inducible factor 1-alpha	HIF1A	Q16665
Ig gamma-1 chain C region	IGHG1	P01857
Induced myeloid leukemia cell differentiation protein Mcl-1	MCL1	Q07820
Inhibitor of nuclear factor kappa-B kinase subunit alpha	CHUK	O15111
Insulin	INS	P01308
Insulin receptor	INSR	P06213
Insulin receptor substrate 1	IRS1	P35568
Insulin-like growth factor 1 receptor	IGF1R	P08069
Insulin-like growth factor II	IGF2	P01344
Insulin-like growth factor-binding protein 3	IGFBP3	P17936
Integrin beta-1	ITGB1	P05556
Intercellular adhesion molecule 1	ICAM1	P05362
Interferon gamma	IFNG	P01579
Interferon regulatory factor 1	IRF1	P10914
Interleukin-1 alpha	IL1A	P01583
Interleukin-1 beta	IL1B	P01584
Interleukin-10	IL10	P22301
Interleukin-17B	IL17B	Q9UHF5
Interleukin-2	IL2	P60568
Interleukin-6	IL6	P05231
Interleukin-8	CXCL8	P10145
Interstitial collagenase	MMP1	P03956
Krueppel-like factor 10	KLF10	Q13118
Lengsin	LGSN	Q5TDP6
Leukotriene A-4 hydrolase	LTA4H	P09960
Lipoprotein lipase	LPL	P06858
Liver carboxylesterase 1	CES1	P23141

Target name	Gene Name	UniProt ID
Low-density lipoprotein receptor	LDLR	P01130
Lysozyme	LYZL1	H0YDZ2
Malate dehydrogenase	MDH1	P40925
Malonamidase E2	bll5103	H7C7Y5
Maltase-glucoamylase, intestinal	MGAM	O43451
Matrix metalloproteinase-9	MMP9	P14780
Metal-binding activator 1	MAC1	P35192
Microsomal triglyceride transfer protein large subunit	MTTP	P55157
Microtubule-associated protein 2	MAP2	P11137
Mineralocorticoid receptor	NR3C2	P08235
Mitochondrial dicarboxylate carrier	SLC25A10	Q9UBX3
Mitochondrial uncoupling protein 2	UCP2	P55851
Mitochondrial uncoupling protein 3	UCP3	P55916
Mitogen-activated protein kinase 1	MAPK1	P28482
Mitogen-activated protein kinase 3	MAPK3	P27361
Mitogen-activated protein kinase 8	MAPK8	P45983
Monomeric sarcosine oxidase	soxA	P40873
M-phase inducer phosphatase 2	CDC25B	P30305
mRNA of PKA Catalytic Subunit C-alpha	PRKACA	P17612
mRNA of Protein-tyrosine phosphatase, non-receptor type 1	PTPN1	P18031
Multidrug resistance protein 1	ABCB1	P08183
Multidrug resistance-associated protein 1	ABCC1	P33527
Muscarinic acetylcholine receptor M1	CHRM1	P11229
Muscarinic acetylcholine receptor M2	CHRM2	P08172
Muscarinic acetylcholine receptor M3	CHRM3	P20309
Muscarinic acetylcholine receptor M4	CHRM4	P08173
Mu-type opioid receptor	OPRM1	P35372
Myc proto-oncogene protein	MYC	P01106
Myeloperoxidase	MPO	P05164
NAD(P)H dehydrogenase [quinone] 1	NQO1	P15559
NAD-dependent deacetylase sirtuin-1	SIRT1	Q96EB6
NAD-dependent deacetylase sirtuin-2	SIRT2	Q8IXJ6
NAD-dependent malic enzyme, mitochondrial	ME2	P23368
NADP-dependent malic enzyme, mitochondrial	ME3	Q16798
NADPH--cytochrome P450 reductase	POR	P16435
Neuromodulin	GAP43	P17677
Neuronal acetylcholine receptor protein, alpha-7 chain	CHRNA7	P36544

Target name	Gene Name	UniProt ID
Neuronal acetylcholine receptor subunit alpha-2	CHRNA2	Q15822
Neutrophil cytosol factor 1	NCF1	P14598
NF-kappa-B inhibitor alpha	NFKBIA	P25963
Nicotinate-nucleotide--dimethylbenzimidazole phosphoribosyltransferase	cobT	Q05603
Nitric oxide synthase, endothelial	NOS3	P29474
Nitric-oxide synthase, brain	NOS1	P29475
Nitric-oxide synthase, endothelial	NOS3	P29474
Nuclear factor erythroid 2-related factor 2	NFE2L2	Q16236
Nuclear receptor coactivator 1	NCOA1	Q15788
Nuclear receptor coactivator 2	NCOA2	Q15596
Nuclear receptor subfamily 1 group I member 2	NR1I2	O75469
Nuclear receptor subfamily 1 group I member 3	NR1I3	Q14994
Ornithine aminotransferase, mitochondrial	OAT	P04181
Ornithine carbamoyltransferase, mitochondrial	OTC	P00480
Ornithine decarboxylase	ODC1	P11926
Osteopontin	SPP1	Q3LGB0
Oxidoreductase	HTATIP2	Q9BUP3
Oxysterols receptor LXR-alpha	NR1H3	Q13133
Pancreas/duodenum homeobox protein 1	PDX1	P52945
Pappalysin-1	PAPPA	Q13219
Peptide YY	PYY	P10082
Peptidyl-glycine alpha-amidating monooxygenase	PAM	P19021
Peroxidase C1A	PRXC1A	P00433
Peroxisome proliferator activated receptor gamma	PPARG	P37231
Peroxisome proliferator-activated receptor alpha	PPARA	Q07869
Peroxisome proliferator-activated receptor delta	PPARD	Q03181
Peroxisome proliferator-activated receptor gamma	PPARG	P37231
Phorbol-12-myristate-13-acetate-induced protein 1	PMAIP1	Q13794
Phosphatidylcholine-sterol acyltransferase	LCAT	P04180
Phosphatidylinositol-3,4,5-trisphosphate 3-phosphatase and dual-specificity protein phosphatase PTEN	PTEN	P60484
Phosphatidylinositol-4,5-bisphosphate 3-kinase catalytic subunit, gamma isoform	PIK3CG	P48736
Phospholipase A2	PLA2G1B	P04054
Phospholipase B1, membrane-associated	PLB1	Q6P1J6
Plasminogen	PLG	P00747
Plasminogen activator inhibitor 1	SERPINE1	P05121
Platelet endothelial cell adhesion molecule	PECAM1	P16284

Target name	Gene Name	UniProt ID
Poly [ADP-ribose] polymerase 1	PARP1	P09874
Potassium voltage-gated channel subfamily H member 2	KCNH2	Q12809
Probable E3 ubiquitin-protein ligase HERC5	HERC5	Q9UII4
Procollagen C-endopeptidase enhancer 1	PCOLCE	Q15113
Pro-epidermal growth factor	EGF	P01133
Progesterone receptor	PGR	P06401
Prolyl 3-hydroxylase 1	P3H1	Q32P28
Prolyl 3-hydroxylase 3	P3H3	Q8IVL6
Prolyl 4-hydroxylase subunit alpha-2	P4HA2	O15460
Prostaglandin E synthase	PTGES	O14684
Prostaglandin E2 receptor EP3 subtype	PTGER3	P43115
Prostaglandin G/H synthase 1	PTGS1	P23219
Prostaglandin G/H synthase 2	PTGS2	P35354
Prostatic acid phosphatase	ACP3	P15309
Protein CBFA2T1	RUNX1T1	Q06455
Protein kinase C alpha type	PRKCA	P17252
Protein kinase C beta type	PRKCB	P05771
Protein kinase C delta type	PRKCD	Q05655
Proto-oncogene c-Fos	FOS	P01100
Proto-oncogene tyrosine-protein kinase SRC	SRC	P12931
Protransforming growth factor alpha	TGFA	P01135
Puromycin-sensitive aminopeptidase	NPEPPS	P55786
Putative beta-glucuronidase-like protein SMA3	GUSBP1	Q15486
Pygopus homolog 1	PYGO1	Q9Y3Y4
Pyruvate kinase isozymes M1/M2	PKM	P14618
RAC-alpha serine/threonine-protein kinase	AKT1	P31749
RAF proto-oncogene serine/threonine-protein kinase	RAF1	P04049
Ras association domain-containing protein 1	RASSF1	Q9NS23
Ras GTPase-activating protein 1	RASA1	P20936
Receptor tyrosine-protein kinase erbB-2	ERBB2	P04626
Receptor tyrosine-protein kinase erbB-3	ERBB3	P21860
Retinoblastoma-associated protein	RB1	P06400
Retinoic acid receptor RXR-alpha	RXRA	P19793
Retinol-binding protein 2	RBP2	D6RB89
Rhinovirus coat protein	N/A	P23008
Rhodopsin	RHO	P08100
Runt-related transcription factor 2	RUNX2	Q13950

Target name	Gene Name	UniProt ID
S-adenosylmethionine synthetase isoform type-1	MAT1A	Q00266
S-adenosylmethionine synthetase isoform type-2	MAT2A	P31153
Serine/threonine-protein kinase Chk2	CHEK2	O96017
Serine/threonine-protein kinase D1	PRKD1	Q15139
Serine/threonine-protein kinase mTOR	MTOR	P42345
Serine--pyruvate aminotransferase	AGXT	P21549
Serum paraoxonase/arylesterase 1	PON1	P27169
Signal transducer and activator of transcription 1-alpha/beta	STAT1	P42224
Signal transducer and activator of transcription 3	STAT3	P40763
Sodium channel protein type 5 subunit alpha	SCN5A	Q14524
Sodium-dependent dopamine transporter	SLC6A3	Q01959
Sodium-dependent noradrenaline transporter	SLC6A2	P23975
Sodium-dependent serotonin transporter	SLC6A4	P31645
Solute carrier family 2, facilitated glucose transporter member 2	SLC2A2	P11168
Solute carrier family 2, facilitated glucose transporter member 4	SLC2A4	P14672
Solute carrier family 22 member 5	SLC22A5	O76082
SPARC	SPARC	P09486
Sterol O-acyltransferase 1	SOAT1	P35610
Sterol O-acyltransferase 2	SOAT2	O75908
Sterol regulatory element-binding protein 1	SREBF1	P36956
Stromelysin-1	MMP3	P08254
Succinate dehydrogenase [ubiquinone] flavoprotein subunit, mitochondrial	SDHA	P31040
Succinate semialdehyde dehydrogenase, mitochondrial	ALDH5A1	P51649
Succinyl-CoA ligase [ADP-forming] beta-chain, mitochondrial	SUCLA2	Q9P2R7
Succinyl-CoA:3-ketoacid-coenzyme A transferase 2, mitochondrial	OXCT2	Q9BYC2
Superoxide dismutase [Cu-Zn]	SOD1	P00441
Superoxide dismutase [Mn], mitochondrial	SOD2	P04179
T-cell-specific surface glycoprotein CD28	CD28	P10747
Telomerase protein component 1	TEP1	Q99973
Thioredoxin reductase, cytoplasmic	TXNRD1	Q16881
Thrombin	F2	P00734
Thrombomodulin	THBD	P07204
Tissue factor	F3	P13726
Tissue-type plasminogen activator	PLAT	P00750
T-lymphocyte activation antigen CD80	CD80	P33681
TNF receptor-associated factor 2	TRAF2	Q12933
Transcription factor AP-1	FOS	P01100

Target name	Gene Name	UniProt ID
Transcription factor E2F1	E2F1	Q01094
Transcription factor E2F2	E2F2	Q14209
Transcription factor p65	RELA	Q04206
Transcription factor Sp1	SP1	P08047
Transforming growth factor beta-1	TGFB1	P01137
Transforming growth factor beta-2	TGFB2	P61812
Transient receptor potential cation channel subfamily V member 1	TRPV1	Q8NER1
Triosephosphate isomerase	TPI1	P60174
Trypsin-1	PRSS1	P07477
Trypsin-3	PRSS3	P35030
Tumor necrosis factor	TNF	P01375
Tumor necrosis factor ligand superfamily member 10	TNFSF10	P50591
Tumor necrosis factor receptor superfamily member 10A	TNFRSF10A	O00220
Tumor necrosis factor receptor superfamily member 10B	TNFRSF10B	O14763
Type I iodothyronine deiodinase	DIO1	P49895
Type-1 angiotensin II receptor	AGTR1	P30556
Tyrosine-protein kinase JAK1	JAK1	P23458
Tyrosine-protein phosphatase non-receptor type 1	PTPN1	P18031
UDP-glucuronosyltransferase 1-1	UGT1A1	P22309
Urokinase-type plasminogen activator	PLAU	P00749
Vascular cell adhesion protein 1	VCAM1	P19320
Vascular endothelial growth factor A	VEGFA	P15692
Vascular endothelial growth factor receptor 2	KDR	P35968
Xanthine dehydrogenase/oxidase	XDH	P47989
X-ray repair cross-complementing protein 6	XRCC6	P12956

Table S3 100 Gastrointestinal motility disorders–related genes

Gene name	Protein name
ACHE	Acetylcholinesterase (Yt blood group)
ACTB	Actin, cytoplasmic 1
ALB	Serum albumin
ANO1	Discovered on gastrointestinal stromal tumors protein 1
ATP12A	ATPase, H+/K+ transporting, nongastric, alpha polypeptide
ATP4A	ATPase, H+/K+ exchanging, alpha polypeptide
CALB2	29 kDa calbindin
CALCA	Calcitonin-related polypeptide alpha
CCK	Cholecystokinin
CCKAR	Cholecystokinin receptor type A
CCL28	Mucosae-associated epithelial chemokine
CHAT	Choline O-acetyltransferase
CLCN2	Chloride channel, voltage-sensitive 2
CLDN2	Claudin 2
CRH	Corticotropin releasing hormone
CRHR1	Corticotropin releasing hormone receptor 1
CRHR2	Corticotropin releasing hormone receptor 2
CXCL8	Monocyte-derived neutrophil chemotactic factor
DPP4	Adenosine deaminase complexing protein 2
EDN3	Preproendothelin-3
EDNRB	Endothelin receptor non-selective type
ELAVL3	Paraneoplastic cerebellar degeneration-associated antigen
ELAVL4	ELAV like neuron-specific RNA binding protein 4
FFAR2	G-protein coupled receptor 43
FFAR3	G-protein coupled receptor 41
FGF19	Fibroblast growth factor 19
FOS	FBJ murine osteosarcoma viral oncogene homolog
GAL	galanin/GMAP prepropeptide
GAPDH	Glyceraldehyde-3-phosphate dehydrogenase
GAST	Gastrin
GCG	Glucagon
GDNF	Glial cell line-derived neurotrophic factor
GHRL	Growth hormone-releasing peptide
GHSR	Growth hormone secretagogue receptor type 1
GIP	Glucose-dependent insulinotropic polypeptide

Gene name	Protein name
GPBAR1	G protein-coupled bile acid receptor 1
GPR55	G protein-coupled receptor 55
GRP	Gastrin-releasing peptide
GUCY2C	Guanylate cyclase 2C (heat stable enterotoxin receptor)
HOXB5	Homeobox protein HHO.C10
HRH2	Histamine H2 receptor
HTR2B	5-hydroxytryptamine (serotonin) receptor 2B, G protein-coupled
HTR3A	5-hydroxytryptamine (serotonin) receptor 3A, ionotropic
HTR4	5-hydroxytryptamine (serotonin) receptor 4, G protein-coupled
HTR7	5-hydroxytryptamine (serotonin) receptor 7, adenylate cyclase-coupled
IL10	Cytokine synthesis inhibitory factor
IL1B	Interleukin 1, beta
IL6	B-cell stimulatory factor 2
INS	Insulin
KIT	V-kit Hardy-Zuckerman 4 feline sarcoma viral oncogene homolog
KITLG	Mast cell growth factor
KLB	Klotho beta-like protein
LEP	Obesity factor
MBOAT4	Membrane-bound O-acyltransferase domain-containing protein 4
MLN	Promotilin
MLNR	G-protein coupled receptor 38
MPO	Myeloperoxidase
MYH11	Myosin heavy chain, smooth muscle isoform
MYLK	Myosin light chain kinase, smooth muscle
NOS1	Peptidyl-cysteine S-nitrosylase NOS1
NPR3	Atrial natriuretic peptide clearance receptor
NPSR1	G-protein coupled receptor for asthma susceptibility
NPY	Pro-neuropeptide Y
NR1H4	Nuclear receptor subfamily 1, group H, member 4
NRTN	Neurturin
NTS	Neurotensin/neuromedin N
OCLN	Occludin
OPRM1	Mu-type opioid receptor
OXT	Oxytocin/neurophysin I prepropeptide
PHOX2B	Paired mesoderm homeobox protein 2B
POMC	Corticotropin-lipotropin
PPP1R14A	Protein phosphatase 1, regulatory (inhibitor) subunit 14A

Gene name	Protein name
PPY	Pancreatic polypeptide
PYY	Peptide tyrosine tyrosine
ROCK2	Rho-associated, coiled-coil-containing protein kinase II
S100B	S100 calcium binding protein B
SCN5A	Sodium channel, voltage-gated, type V, alpha subunit
SCT	Secretin
SLC18A3	Solute carrier family 18 (vesicular acetylcholine transporter), member 3
SLC6A4	Solute carrier family 6 (neurotransmitter transporter), member 4
SMTN	Smoothelin
SNCA	Synuclein, alpha (non A4 component of amyloid precursor)
SOX10	SRY (sex determining region Y)-box 10
SST	Growth hormone release-inhibiting factor
TAC1	Tachykinin, precursor 1
TACR1	Tachykinin receptor 1
TACR2	Neurokinin A receptor
TACR3	Neurokinin B receptor
TH	Tyrosine 3-monooxygenase
TLR4	Toll-like receptor 4
TNF	Tumor necrosis factor ligand superfamily member 2
TPH1	Tryptophan hydroxylase 1
TRPA1	Transient receptor potential cation channel, subfamily A, member 1
UCHL1	Ubiquitin carboxyl-terminal esterase L1 (ubiquitin thiolesterase)
UCN	Urocortin
UCN3	Urocortin III
VIP	Vasoactive intestinal peptide
VIPR1	Pituitary adenylate cyclase-activating polypeptide type II receptor
VR1	Transient receptor potential cation channel, subfamily V, member 1
WRB	Tail-anchored protein insertion receptor WRB
